# Polypharmacy and Dose Adjustment in Chronic Kidney Disease: A Cross-Sectional Study

**DOI:** 10.14740/jocmr6444

**Published:** 2026-02-28

**Authors:** Jakob Mokros, Igor Matyukhin, Oliver Ritter, Daniel Patschan

**Affiliations:** a Department of Internal Medicine–Cardiology, Angiology, Nephrology and Pulmology, Brandenburg University Hospital, Brandenburg Medical School (Theodor Fontane), Brandenburg, Germany; b Faculty of Health Sciences (FGW), Joint Faculty of University of Potsdam, Brandenburg Medical School Theodor Fontane and Brandenburg Technical University Cottbus-Senftenberg, Cottbus, Germany

**Keywords:** CKD, Polypharmacy, Dose-adjustment

## Abstract

**Background:**

Chronic kidney disease (CKD) is a significant global health issue, primarily due to the rise in diabetes mellitus. This study aims to analyze the medications used in CKD patients with varying severity, focusing on dose adaptation.

**Methods:**

This was a retrospective observational analysis of patients with CKD from various causes. CKD staging followed the 2024 Kidney Disease: Improving Global Outcomes (KDIGO) guidelines, and all medications given during the study were recorded, including documentation of dose adjustments due to reduced kidney function.

**Results:**

The study included 106 CKD patients. A total of 209 active medications were examined, with an average of 11.2 ± 4.8 substances used per patient. The average number of medications did not differ significantly across CKD stages. Dose adjustments for reduced kidney function were required in 40.19% of patients, who received an average of 5.4 ± 4.6 medications requiring dose reduction, with appropriate adjustments made for 4.6 ± 2.2 substances on average.

**Conclusions:**

The study found that polypharmacy is present in all stages of CKD, and the significant rate of dose adjustments suggests that physicians are aware of the need to manage medications for CKD patients.

## Introduction

In Central Europe, approximately 15% of all adults are affected by chronic kidney disease (CKD) [1]. The prevalence of CKD has risen in recent years, primarily due to the global increase in diabetes mellitus, which is the most common isolated cause of CKD [2]. Additionally, the aging population in Central Europe contributes to this trend, as cumulative morbidity tends to rise with age. CKD patients face a heightened risk of cardiovascular complications [3–5], a connection that was convincingly established by Go et al in 2004 [6]. Beyond cardiovascular issues, CKD patients also experience bone metabolism disorders, renal anemia, secondary metabolic acidosis, as well as dermatological, gastrointestinal, and potentially neurological manifestations [7, 8].

It is evident that CKD necessitates consistent utilization of diverse types of medications. Chronic kidney disease mineral bone disorder (CKD-MBD) often requires the use of active vitamin D metabolites and various oral phosphate binders, particularly in cases of high turnover osteopathy [9]. In some cases, oral phosphate binders may require higher doses (e.g., calcium acetate, two tablets three times with meals). The high blood pressure that frequently occurs in CKD can typically only be managed with combinations of three, four, or more active medications. In cases of established cardiovascular disease, the standard of care often involves the regular use of acetylsalicylic acid, statins, and oral antidiabetics. Therefore, individuals requiring dialysis (CKD stage 5D), for example, often have to take 10 or more prescription medications on a regular basis [10]. The challenges this poses to patient compliance are not addressed in this context. Regardless of compliance, the risk of drug interactions increases with the degree of polypharmacy. Finally, the decline in renal excretory function necessitates adjustments to medication dosages or the use of alternative substances, as the reduction in glomerular filtration rate (GFR) increases the risk of accumulation. This is particularly relevant for medications with higher water solubility. Medications that strongly bind to plasma proteins are less likely to be eliminated through the kidneys. Significant urinary excretion of these protein-bound drugs typically occurs only when there are underlying health issues, such as nephrotic syndrome. However, it should be noted that certain drugs with significant plasma protein binding are nevertheless eliminated renally, such as certain penicillins. In such cases, tubular secretion may be the primary route of elimination [11]. In any case, physicians are confronted with various challenges when treating CKD patients with regard to the correct administration of drug therapies.

The aim of this retrospective survey was to analyze drug therapy among patients with CKD, with particular emphasis on the average number of medications used at each CKD stage and the frequency of estimated glomerular filtration rate (eGFR)-guided dosage adjustments when indicated.

## Materials and Methods

### Design, ethics and data sources

The current investigation is a retrospective observational cohort study. The data were collected as part of a project that was reviewed and approved by the Ethics Committee of the Brandenburg Medical School (204052024-BO-E-RETRO). Due to the retrospective and anonymous nature of the study, it was not necessary to obtain written consent from the patients. The requirements of the Declaration of Helsinki were taken into account. All data were collected between January 21, 2017, and September 13, 2022. The study was conducted as a single-center evaluation at Brandenburg University Hospital, Brandenburg Medical School. Patients from all specialist disciplines at the hospital were included, provided they met the inclusion criteria. All collected data were extracted from the central database of Brandenburg University Hospital (CGM MEDICO^®^). The database systematically documents all information associated with individual treatment cases, encompassing physicians’ and nurses’ notes, detailed examination results, laboratory parameters, daily medication records, and discharge summaries.

### Patients

The patients included in the study were identified using code lists provided by the medical controlling department. The inclusion criteria were as follows: patients had to be aged 18 years or older and have CKD of various etiologies and variable stages according to Kidney Disease: Improving Global Outcomes (KDIGO) 2024 [8]. The exclusion criteria were as follows: patients were pregnant, had acute kidney injury according to KDIGO 2012 [12] without pre-existing CKD, or were at terminal disease stages requiring palliative care. After identification, the CKD diagnosis was re-evaluated based on the eGFR (Chronic Kidney Disease Epidemiology Collaboration (CKD-EPI) formula) in accordance with the KDIGO guideline from 2024 [8]. Urinary albumin excretion could not be consistently taken into account, as the corresponding values were missing in too many cases. The indications for hospital admission were assigned to one of 11 categories: heart failure, pneumonia, non-respiratory infection including sepsis, metabolic disorder (assigned to this category even if electrolyte disturbances were the reason for admission), acute kidney injury with pre-existing CKD, end-stage CKD (stage 5D), malignant disorder, liver cirrhosis/liver failure, trauma, and others. The eGFR (CKD-EPI) was also documented upon admission and prior to discharge from the hospital, as were the serum concentrations of the following parameters at the time of admission: sodium, potassium, calcium, phosphate, low-density lipoprotein (LDL) cholesterol, high-density lipoprotein (HDL) cholesterol, and total cholesterol. Hemoglobin concentration and glycated hemoglobin (HbA1c) were also recorded.

### Drug therapy and dosing evaluation

All medications administered to all patients included in the study were documented at the time of inclusion. This applied to both oral and parenteral therapeutic agents. Decisions concerning “appropriate” or “inappropriate” dosing were determined exclusively according to the recommendations provided by the respective manufacturers, which are established with a primary focus on patient safety. The lowest eGFR recorded during hospitalization, as calculated by the CKD-EPI method [13], served as the criterion for these decisions.

### Statistics

The following application was used to perform statistical analyses: Wizard for MacOS (version 2.0.19). The results of the survey were given either as percentages or absolute numbers or as mean values ± standard deviation. Comparisons between two or more groups were made using the Chi-square test in the case of categorical data; non-categorical data were compared using either the *t*-test or the Mann–Whitney test (normally distributed or non-normally distributed) if there were two groups. For more than two groups, analysis of variance (ANOVA) or Kruskal–Wallis tests were used (normally distributed or non-normally distributed). Correlation analyses were conducted using Pearson’s method. A P value of less than 0.05 was considered statistically significant.

## Results

### Patients

A total of 106 CKD patients were included in the study. Among the participants, 50 (47.2%) were female and 56 (52.8%) were male. The mean age was 77 years, with a standard deviation of 10.9 years. The average length of hospital stay was 13.2 days, with a standard deviation of 11.7 days. The following CKD etiologies were identified: diabetic/hypertensive nephropathy (42.5%); hypertensive nephropathy (32.1%); diabetic nephropathy (14.2%); unknown (9.4%); glomerulonephritis (0.9%); and others (0.9%). The distribution of CKD stages according to KDIGO 2024 [8] was as follows: stage 2 (9.6%); stage 3 (a and b, (53.8%); stage 4 (15.4%); and stage 5 (non-dialysis (ND) and dialysis (D), 21.2%). The four CKD stages significantly differed in terms of the mean age of the patients: stage 2 (80 ± 3 years); stage 3 (78.2 ± 1.4 years); stage 4 (81.5 ± 2.1 years); stage 5 (ND/D) (68.5 ± 2.1 years) (P < 0.001). Patients were hospitalized for the following reasons, listed in order of decreasing frequency: heart failure (40.6%); pneumonia (19.8%); non-respiratory infections including sepsis (9.4%); metabolic disorders (includes disorders of the glucose metabolism, the thyroid gland and electrolyte disorders (5.7%)); acute kidney injury with pre-existing CKD (4.7%); vascular disorders (4.7%); other (4.7%); CKD stage 5D (3.8%); malignant disorders (2.8%); liver cirrhosis/liver failure (1.9%); and trauma (1.9%). Table 1 summarizes all patient characteristics.

**Table 1:** Patient Characteristics at the Time of Study Inclusion

Variable	Result
Gender (females/males, %)	47.2/52.8
Age (years)	77 ± 10.9
In-hospital therapy (days)	13.2 ± 11.7
CKD etiology n (%)	
Diabetic/hypertensive nephropathy	45 (42.5)
Hypertensive nephropathy	34 (32.1)
Diabetic nephropathy	15 (14.2)
Glomerulonephritis	1 (0.9)
Other	1 (0.9)
Unknown	10 (9.4)
CKD stage (KDIGO 2024) n (%)	
2	10 (9.6)
3	56 (53.8)
4	16 (15.4)
5 (ND/D)	22 (21.2)
Reason for hospitalization n (%)	
Heart failure	43 (40.6)
Pneumonia	21 (19.8)
Non-respiratory infections including sepsis	10 (9.4)
Metabolic disorder	6 (5.7)
Acute kidney injury (with pre-existing CKD)	5 (4.7)
Vascular disorder	5 (4.7)
Chronic kidney disease	4 (3.8)
Malignant disorder	3 (2.8)
Liver cirrhosis/liver failure	2 (1.9)
Trauma	2 (1.9)
Other	5 (4.7)
Laboratory findings on admission	
eGFR (mL/min)	31.6 ± 17.6
Serum sodium (mmol/L)	136.4 ± 5.9
Serum potassium (mmol/L)	4.4 ± 0.8
Serum calcium (mmol/L)	1.8 ± 0.4
Serum phosphate (mmol/L)	1.4 ± 0.6
Hemoglobin (g/L)	111.9 ± 22.2
Morbidities n (%)	
Arterial hypertension	80 (75.5)
Coronary artery disease	46 (43.3)
Chronic heart failure	34 (32.1)
Obesity	29 (27.3)
Diabetes mellitus	58 (54.7)
Chronic obstructive pulmonary disease	23 (19.4)
History of neoplasia	18 (17)
Smoking	12 (11.3)

CKD: chronic kidney disease; KDIGO: Kidney Disease: Improving Global Outcomes; ND: non-dialysis; D: dialysis; eGFR: estimated glomerular filtration rate.

### Characteristics of drug therapy in the overall CKD cohort

At the time of analysis, an average of 11.2 ± 4.8 individual medications were being used across the entire cohort. All types of applications, including oral, intravenous, and topical, were considered. There were no differences observed between women and men concerning the number of substances used: women 11.3 ± 0.6, men 11.1 ± 0.6 (P = 0.91). The number of medications taken did not correlate with age (P = 0.67). The number also did not correlate with the length of hospital stay (P = 0.13). The patients were categorized based on the number of medications used (quantity categories): fewer than four medications (n = 6), four to nine medications (n = 31), and 10 or more medications (n = 69), with the latter group representing 65% of the total cohort. A total of 209 individual active medications were examined in the study. Based on the number of substances used per group, the following results were obtained (in descending order of frequency): metabolic/hormonal substances/vitamins (20.09%); hemodynamic/diuretic substances (18.7%); antimicrobial substances (11%); psychotropic substances (8.13%); pulmonary substances (7.65%); neuromuscular substances (7.17%); immunomodulatory/antiallergic substances (5.74%); antithrombotic/anticoagulatory substances (5.26%); analgesics (5.26%); antidiabetics (2.87%); antiarrhythmics (0.47%); other (3.34%) (Table 2).

**Table 2:** Substance Groups Including All Individual Substances

Group	Substances	N (%) of all substances applied
Hemodynamic/diuretic	Adaption required: chlorthalidone, enalapril, empagliflozin, eplerenone, hydrochlorothiazide, indapamide, lercanidipine, lisinopril, moxonidine, naftidrofuryl oxalate, nebivolol, olmesartan, propranolol, ramipril, sacubitril, spironolactone, telmisartan, torsemide	39 (18.7%)
	Adaption not required: amlodipine, bisoprolol, candesartan, carvedilol, clonidine, dihydropyridine derivative, doxazosin, felodipine, furosemide, glyceryl trinitrate, isosorbide mononitrate, losartan, metoprolol, minoxidil, molsidomine, nitrendipine, noradrenaline, urapidil, valsartan, verapamil, xipamide	
Antiarrhythmic	Adaption not required: amiodarone	1 (0.47%)
Antithrombotic/anticoagulatory	Adaption required: acetylsalicylic acid, apixaban, edoxaban, enoxaparin, heparin, phenprocoumon, rivaroxaban	11 (5.26%)
	Adaption not required: clopidogrel, digitoxin, prasugrel, ticagrelor	
Pulmonary	Adaption required: fenoterol, salbutamol	16 (7.65%)
	Adaption not required: acetylcysteine, aclidinium, ambroxol, beclomethasone, budesonide, formoterol, glycopyrronium bromide, indacaterol, ipratropium bromide, montelukast, olodaterol, salmeterol, theophylline, tiotropium	
Antimicrobial	Adaption required: acyclovir, amphotericin B, cefuroxime, ciprofloxacin, clarithromycin, co-trimoxazole, erythromycin, flucloxacillin, gentamicin, levofloxacin, meropenem, piperacillin, valganciclovir, vancomycin	23 (11%)
	Adaption not required: ampicillin + sulbactam, azithromycin, cefpodoxime, ceftazidime, ceftriaxone, linezolid, metronidazole, nystatin, rifaximin	
Immunomodulatory/antiallergic	Adaption required: mesalamine	12 (5.74%)
	Adaption not required: adalimumab, dexamethasone, everolimus, fexofenadine, fluticasone, hydrocortisone, methylprednisolone, mycophenolic acid, prednisolone, rituximab, tofacitinib	
Antidiabetics	Adaption required: glimepiride, metformin, semaglutide, sitagliptin, vildagliptin	6 (2.87%)
	Adaption not required: insulin	
Metabolic/hormonal/vitamins	Adaption required: alendronate, allopurinol, cholecalciferol, epoetin alfa, magnesium, Nutriflex^®^ Peri, pravastatin, rosuvastatin, sevelamer, simvastatin, sodium bicarbonate, sodium polystyrene sulfonate	42 (20.09%)
	Adaption not required: alfacalcidol, atorvastatin, bicalutamide, calcium, calcium carbonate, calcitriol, cinacalcet, darbepoetin alpha, denosumab, Dreisavit^®^ (water soluble vitamins), dutasteride, exemestane, ezetimibe, febuxostat, Ferrlecit^®^, Ferro Sanol^®^, finasteride, folic acid, Fosrenol^®^, glucose, hyaluronic acid, levothyroxine, potassium, Renavit (water soluble vitamins), Tardyferon, thiamazol, vitamin B1, vitamin B6, vitamin B12, Vitarenal^®^ (water soluble vitamins)	
Psychotropic	Adaption required: amitriptyline, citalopram, diazepam, midazolam, mirtazapine, opipramol, piracetam, risperidone	17 (8.13%)
	Adaption not required: benperidol, doxepin, lorazepam, melatonin, melperone, paroxetine, pipamperon, trimipramine, zopiclone	
Analgesic	Adaption required: ketoprofen, morphine, oxycodone	11 (5.26%)
	Adaption not required: buprenorphine, fentanyl, metamizole, naloxegol, paracetamol, tilidine, tramadol, xylometazoline	
Gastrointestinal	Adaption required: Dropizol^®^, metoclopramide, omeprazole, pantoprazole	9 (4.3%)
	Adaption not required: espumisan, granisetron, lactulose, macrogol, saccharomyces cerevisiae	
Neuromuscular	Adaption required: gabapentin, galantamine, lamotrigine, levetiracetam, levodopa, Madopar^®^, pramipexole, pregabalin, propiverine, venlafaxine	15 (7.17%)
	Adaption not required: darifenacin, propofol, quetiapine, remifentanil, valproate	
Other	Adaption not required: Helixor^®^, latanoprost, letrozole, olopatadine, osimertinib, tamsulosin, timolol	7 (3.34%)

### Drug therapy in different CKD stages

In patients with CKD stages 2, 3, 4, and 5 (ND and D), the average number of medications used were as follows: stage 2 (10.9 ± 1.5); stage 3 (10.8 ± 0.6); stage 4 (10.3 ± 0.9); and stage 5 (ND and D) (12.8 ± 1). Statistical significance was not achieved (P = 0.34). Similarly, there was no significant difference between the stages regarding the aforementioned quantity categories. The distribution was as follows: stage 2—< 4: 0%; 4–9: 30%; 10 or more: 70%; stage 3—< 4: 8.9%; 4–9: 32.1%; 10 or more: 58.9%; stage 4—< 4: 6.2%; 4–9: 31.2%; 10 or more: 62.5%; stage 5 (ND/D)—< 4: 0%; 4–9: 18.2%; 10 or more: 81.8% (P = 0.51). When analyzing only women or men separately, no significant differences were found between stage 5 (ND/D) and all other stages combined regarding the total number of medications used: women—stage 5 (ND/D), 14.6 ± 1.5 vs. all other stages, 10.3 ± 0.7; men—stage 5 (ND/D), 11 ± 1.1 vs. all other stages, 11.1 ± 0.7 (P = 0.06). The quantity categories were distributed as follows: women—stage 5 (ND/D), < 4: 0%; 4–9: 18.2%; 10 or more: 81.8% vs. all other stages, < 4: 7.7%; 4–9: 33.3%; 10 or more: 59%); men—stage 5 (ND/D), < 4: 0%; 4–9: 18.2%; 10 or more: 81.8% vs. all other stages, < 4: 7%; 4–9: 30.2%; 10 or more: 62.8%).

### Dose adjustment

Among these, 84 substances (40.19%) required dose adjustment in cases of reduced renal excretory function. On average, patients received 5.4 ± 4.6 medications for which a dose reduction was indicated due to CKD. When quantifying the number of substances that need to be adjusted for each stage of CKD, the following results were obtained: stage 2, 4 ± 0.6; stage 3, 5.6 ± 0.3; stage 4, 4.9 ± 0.3; stage 5 (ND/D), 6 ± 0.5 (P = 0.1). Once again, there was no difference between women and men in the number of medications (women, 5.4 ± 0.3; men, 5.4 ± 0.3; P = 0.86), nor was there any correlation between the number of active ingredients to be adapted and age (P = 0.87). An appropriate dose adjustment was made for an average of 4.6 ± 2.2 medications with the corresponding indication. Table 3 lists differences in the frequency of use of substances requiring dose adjustment between the different stages of CKD.

**Table 3:** Differences in the Frequency of Use of Substances Requiring Dose Adjustment Between the Different Stages of CKD

Medication	P value
ASS	0.74
Acyclovir	0.83
Alendronate	0.72
Allopurinol	0.14
Amitriptyline	0.83
Amphotericin B	0.83
Apixaban	0.55
Cefuroxime	0.62
Chlorthalidone	0.44
Ciprofloxacin	0.13
Citalopram	0.91
Clarithromycin	0.62
Cholecalciferol	0.42
Co-trimoxazole	0.05
Diazepam	0.83
Dropizol	0.28
Enalapril	0.03
Edoxaban	0.34
Enoxaparin	0.84
Empagliflozin	0.31
Eplerenone	0.13
Epoetin alfa	< 0.001
Erythromycin	0.28
Fenoterol	0.9
Flucloxacillin	0.83
Glimepiride	0.83
Gabapentin	0.72
Gabrilen (ketoprofen)	0.83
Galantamine	0.83
Gentamicin	0.83
Heparin (high-molecular weight heparin)	< 0.001
Hydrochlorothiazide	0.5
Indapamide	0.83
Lamotrigine	0.83
Lercanidipine	0.24
Levetiracetam	0.24
Levodopa	0.72
Levofloxacin	0.7
Lisinopril	0.13
Madopar	0.02
Magnesium	0.19
Meropenem	0.13
Mesalamine	0.28
Metformin	0.016
Metoclopramide	0.7
Midazolam	0.28
Mirtazapine	0.55
Morphine	0.4
Moxonidine	0.13
Naftidrofuryl oxalate	0.83
Nebivolol	0.07
Nutriflex Peri	0.83
Olmesartan	0.21
Omeprazole	0.62
Opipramol	0.28
Oxycodone	0.63
Pantoprazole	0.06
Phenprocoumon	0.2
Piperacillin	0.02
Piracetam	0.83
Pramipexole	0.28
Pravastatin	0.72
Pregabalin	0.55
Propiverine	0.83
Propranolol	0.83
Ramipril	0.1
Risperidone	0.44
Rivaroxaban	0.49
Rosuvastatin	0.59
Sacubitril	0.62
Salbutamol	0.44
Semaglutide	0.83
Sevelamer	0.009
Simvastatin	0.69
Sitagliptin	0.08
Sodium bicarbonate	< 0.001
Sodium polystyrene sulfonate	0.38
Spironolactone	0.17
Telmisartan	0.7
Torsemide	0.37
Valganciclovir	0.28
Vancomycin	0.52
Venlafaxine	0.25
Vildagliptin	0.83

A P value of less than 0.05 indicates that a particular drug has not been used with comparable frequency in all stages of CKD. CKD: chronic kidney disease; ASS: Acetylsalicylic acid.

## Discussion

Although our study was retrospective in design, it yielded some surprising results. First, no difference was found in the total number of drugs used between CKD stages 2–5 (ND/D). The same applied to the total number of medications with indications for dose adjustment in CKD. Additionally, there were no correlations between age, length of hospital stay, and the number of drugs used. Finally, the dose was appropriately adjusted in the vast majority of cases. Regarding dose adjustment, this survey captures patient care at a single point in time, recording and evaluating each individual’s medication according to the inclusion criteria. However, it does not account for any dose adjustments made during inpatient therapy, as daily monitoring of medication would have been required.

Polypharmacy is not an issue that primarily affects individuals with impaired kidney function or CKD. In principle, it affects older, multimorbid patients. In a clinic-based study, Atak et al [14] evaluated potentially inappropriate medications (PIMs) in 104 adults aged > 65, using the Beers Criteria [15]. The sample included older men and women (mean ages: about 77–79 years; P = 0.30). Only 18 patients (17%) had no increased risk from PIMs; the remaining patients had PIMs associated with increased acute kidney injury risk (n = 30), fall risk (n = 30), lack of safety/efficacy data (n = 20), bleeding risk (n = 12), or amplified neurological side effects (n = 5). The number of Beers-related risks correlated positively with both the number of medications used (r = 0.366, P < 0.001) and the number of comorbidities (r = 0.312, P = 0.001). Another study [16] evaluated 50 patients aged 65 years or older with type 2 diabetes to examine the relationship between glycemic control and polypharmacy. Patients were classified as poorly controlled (HbA1c > 7.5, n = 27) or well-controlled (HbA1c ≤ 7.5, n = 23). Poorly controlled patients had significantly more daily medications (P < 0.001), more comorbidities (P = 0.001), and more medication-related risks per the Beers Criteria (P = 0.02). HbA1c correlated positively with number of medications (r = 0.40, P = 0.004), comorbidities (r = 0.28, P = 0.04), and Beers Criteria risks (r = 0.31, P = 0.014).

The issue of polypharmacy in CKD on the other hand has been addressed in numerous studies, whereas the topic of dose adjustment has not received as much attention. Adjeroh et al [10] conducted an analysis of ND CKD patients and categorized polypharmacy into three groups based on the number of medication classes: four or fewer (minor polypharmacy), five to nine (major polypharmacy), and 10 or more (hyperpolypharmacy). The study considered data from 339,883 participants in the Medical Expenditure Panel Survey (MEPS), identifying 649 individuals with CKD who met distinct inclusion criteria. The average age of CKD patients was 61.55 years, with a significant majority (76.89%) experiencing major or hyperpolypharmacy. The demographic breakdown showed that 62.89% were White, 20.66% Black, 10.32% Hispanic, and 6.14% from other races. Patients with three or more comorbidities exhibited higher rates of polypharmacy, with hypertension being the most common comorbidity (84.82%). Psychiatric illnesses, including depression, serious mental disorders (SMDs), and anxiety, were significantly associated with polypharmacy. The most frequently prescribed medications for ND CKD patients included antihyperlipidemic agents, beta-blockers, antidiabetic agents, analgesics, and diuretics. Notably, patients with hyperpolypharmacy reported the lowest health-related quality of life (HRQoL) scores, with significant differences observed across various demographic factors, comorbidities, and psychiatric conditions. In adjusted models, major polypharmacy and hyperpolypharmacy were linked to lower physical component summary (PCS) and mental component summary (MCS) scores, with depression, lack of physical activity, and public insurance showing the strongest negative associations with HRQoL. The findings underscore the need for careful management of polypharmacy among CKD patients to improve their overall well-being. In 2024, Oosting et al [17] published a comprehensive meta-analysis on polypharmacy in CKD. The literature search identified 15,078 articles, with 127 studies ultimately included in the review. The studies varied in design, with a total population of 547,422 patients, predominantly from Asia and Europe. The median patient age was 64 years, and 56% were men. The prevalence of polypharmacy (defined as taking five or more medications) was 82%, higher in patients undergoing dialysis (89%) or kidney transplants (87%) compared to those with CKD stages 3–5 (68%). Hyperpolypharmacy (10 or more medications) was noted in 40% of patients, particularly in dialysis patients (55%). The average number of prescribed medications was 9.7, with the highest in North America. Polypharmacy was linked to increased all-cause mortality, decline in kidney function, and higher hospitalization rates, while also negatively impacting quality of life and medication adherence. The study highlighted a significant medication burden among CKD patients, with a noted association between the number of medications and adverse drug reactions. Finally, in a more recent study, Nakamura et al [18] analyzed recipients of public assistance with advanced CKD. The study involved 626 patients, of whom 592 were included in the analysis, comprising 56 (9.5%) recipients and 536 (90.5%) non-recipients of public assistance. The mean age of the cohort was 69.6 years, with 59.3% being male and an average eGFR of 10.8 mL/min/1.73 m^2^. Recipients of public assistance exhibited higher prevalence rates of current smoking, diabetes, polypharmacy, and social isolation compared to non-recipients. Regarding the presentation of kidney replacement therapy (KRT) options, 70.3% of non-recipients received such information, while only 51.8% of recipients did. Multivariable logistic regression analysis revealed that recipients of public assistance were significantly less likely to receive a presentation of KRT options (adjusted odds ratio (aOR), 0.31). Additionally, recipients of public assistance were more likely to be on a higher number of medications, specifically receiving ≥ 10 (aOR, 1.92) and ≥ 15 (aOR, 2.78) types, but not more likely to be on ≥ 5 types (aOR, 0.69). Sensitivity analysis confirmed that recipients had a higher likelihood of moving up in medication categories (aOR, 1.82). Overall, the findings highlight disparities in healthcare access and medication management between recipients and non-recipients of public assistance.

Although stages 5 ND and 5 D of CKD were analyzed together in our study, previous literature suggests that a higher prevalence of polypharmacy is expected in the later stages of CKD compared to stage 2. The lack of correlation with age in our findings is also unexpected. The minimum age in our sample was 44 years, the maximum was 95 years, and the median age was 78 years. One possible explanation for the absence of differences between stages could be the age distribution within the stages themselves. The mean age in stages 2 through 4 was approximately 80 years, while in stage 5 (ND/D), it was 68 years. This lower average age in the terminal stage may reflect the significantly increased mortality risk associated with progressive CKD. As previously demonstrated in 2004 [6], the risk of mortality in CKD rises disproportionately with decreasing GFR and increasing proteinuria. Thus, the lower average age observed in stage 5 (ND/D) may be a consequence of the markedly higher mortality rate at this stage. Additionally, it is important to note a key distinction between our data and studies like the one conducted by Adjeroh et al [10]. Their analysis drew on information from over 330,000 individuals using the MEPS, identifying 649 people with CKD—making their sample six times larger than ours. Similarly, the meta-analysis by Oosting et al, mentioned above, reviewed data from more than 540,000 patients across 127 selected studies, resulting in an evaluated group that is 5,000 times bigger than ours. Their findings indicated that polypharmacy was more common among those requiring dialysis or transplants compared to patients with milder forms of CKD. Given this, it is likely that the relatively small size of our cohort affects our results regarding stage-related polypharmacy.

A rather surprising finding is the high rate of dose adjustments. On average, 4.6 out of 5.2 substances that required adjustments also underwent dose modifications. This indicates a notable level of medical awareness regarding the challenges posed by altered pharmacokinetics in CKD. In other areas of nephrological medicine, a notably lower quality of care has been identified. For instance, Adejumo et al reported in 2017 that only 1.2% of all physicians who do not specialize in nephrology possessed what was considered “good” knowledge of acute kidney injury [19]. The patients included in our study were recruited from all specialist departments within the hospital, including various surgical units. Consultations with nephrology specialists occur infrequently, typically only on a sporadic basis.

### Limitations

First, the retrospective, monocentric design presents a limitation. Retrospective studies generally allow for conclusions with limited generalizability, primarily due to often incomplete datasets. In our study, for instance, we were unable to include the now guideline-recommended parameter urinary albumin-to-creatinine ratio (UACR), because measurements were frequently not available. Electrolyte measurement results were also not available from all individuals. Additionally, the monocentric design may be associated with selection bias, as the patient cohort treated at the Brandenburg University Hospital is not representative of the entire country or even larger regions. Another limitation is the failure to consider kidney function parameters or proteinuria before or after the hospital stay. Consequently, the staging of CKD may have been inaccurately performed in some cases. Also, the age distribution across the stages of CKD must be revisited. Under prospective conditions, the CKD subgroups would likely have been formed more uniformly. The lower average age observed in stage 5 may somewhat distort the results. Finally, the assessor of the medication was not blinded, which means reviewer-associated bias could have been introduced.

## Data Availability

The data supporting the findings of this study are available from the corresponding author upon reasonable request.
